# Exploring applications of blockchain in healthcare: road map and future directions

**DOI:** 10.3389/fpubh.2023.1229386

**Published:** 2023-09-15

**Authors:** Yuvraj Singh, M. A. Jabbar, Shishir Kumar Shandilya, Olena Vovk, Yaroslav Hnatiuk

**Affiliations:** ^1^School of Computing Science and Engineering, VIT Bhopal University, Bhopal, Madhya Pradesh, India; ^2^Department of Computer Science and Engineering (AI&ML), Vardhaman College of Engineering, Hyderabad, Telangana, India; ^3^Department of Artificial Intelligence Systems, Lviv Polytechnic National University, Lviv, Oblast, Ukraine

**Keywords:** blockchain, distributed public ledger, healthcare, electronic health record, personal health record, medical health insurance

## Abstract

Blockchain technology includes numerous elements such as distributed ledgers, decentralization, authenticity, privacy, and immutability. It has progressed past the hype to find actual use cases in industries like healthcare. Blockchain is an emerging area that relies on a consensus algorithm and the idea of a digitally distributed ledger to eliminate any intermediary risks. By enabling them to trace data provenance and any changes made, blockchain technology can enable different healthcare stakeholders to share access to their networks without violating data security and integrity. The healthcare industry faces challenges like fragmented data, security and privacy concerns, and interoperability issues. Blockchain technology offers potential solutions by ensuring secure, tamper-proof storage across multiple network nodes, improving interoperability and patient privacy. Encrypting patient data further enhances security and reduces unauthorized access concerns. Blockchain technology, deployed over the Internet, can potentially use the current healthcare data by using a patient-centric approach and removing the intermediaries. This paper discusses the effective utilization of blockchain technology in the healthcare industry. In contrast to other applications, the exoteric evaluation in this paper shows that the innovative technology called blockchain technology has a major role to play in the existing and future applications of the healthcare industry and has significant benefits.

## 1. Introduction

Blockchain technology has been around for at least 20 years. It wasn't until recently that academics and businesses began to consider it strongly. This paper aims to highlight blockchain technology's existing and future applications in the healthcare industry through a thorough analysis. The authors wanted to highlight projects from the scientific and commercial sectors. This article may alternatively be seen as a concise summary and annotation of the idea behind the potential connections between healthcare and cryptocurrency and blockchain technology.

The blockchain's core value is closely related to trust and decentralization. A distributed database called blockchain supports transactions between unreliable entities, instead of having banks or another brokerage firm it acts as a middleman as is the case with traditional transactions, people and organizations may deal directly with one another. Because of this, it is anticipated that the blockchain will alter how people conduct international payments. It is crucial to investigate the potential benefits that this new technology might bring to the healthcare industry. It is clear that instead of just financial transactions, this effort will focus on healthcare data applicability and operations in general.

One of the biggest sectors, healthcare accounts for more than 10% of the GDP of developed nations. The expense of providing effective healthcare services is rising, and patient data is fragmented which calls for the need to protect patient data and deliver effective services to be expanding whereas patient data is scattered, and sharing this private information may occasionally be subject to the practice of authorization management. Data is sometimes unavailable and inaccessible; all such problems in healthcare may be resolved with the help of blockchain. Distributed ledger technology is the foundation of blockchain technology, where transactions occur amongst peers rather than through a central authority. Decentralized transactions will be used throughout none of the entities can change any of the transactions once they have been added since they are all immutable which offers security and privacy for the transactions. A single location may be used to store patient data as a result, diagnosing a patient will be simple. IoT and blockchain can be utilized for real-time patient monitoring. As patient claims are properly monitored by health insurance companies, records are kept in a ledger that cannot be changed after it has been added. Blockchain has a wide variety of features that allow its implementation in a variety of applications. Decentralized storage and authentication are one of the key characteristics of blockchain. There are three main types of blockchain: public, private, and federated. Table 1 lists different types of blockchains. Anyone may participate in and validate public transactions on a public blockchain. The public is in charge of maintaining this kind of blockchain. Public blockchains include, for example, (1) Ethereum, (2) Bitcoin, (3) Bit shares, (4) Waves, and (5) Dash. Government agencies are in charge of maintaining private blockchains. Transactions are internally vetted and not accessible to the public. The consortium maintains federated blockchains, the third form of blockchain. This blockchain may or may not make transactions public. Federated blockchains include B3i, EWF, and Corda R3 as examples.

**Table 1:** Blockchain types.

**S.no**	**Blockchain type**	**Important property**
1	Public blockchain	a) Maintained by the Public b) Anyone can participate and validate the transactions
2	Private blockchain	a) Maintained by Government Organizations b) Transactions are not public
3	Federated blockchain	a) Maintained by consortium b) Transactions may not be public

Blockchain has started to infiltrate a variety of different industries over the past few years, including finance and banking, capital markets, trade finance, business, real estate, media, government organizations, etc. The area where blockchain has enormous potential is healthcare. Data security, data access, data sharing, and interoperability are the top needs in the healthcare industry. Confidentiality and security are fundamental needs of the healthcare sector to protect patient medical information. In this digital age, cloud storage has become the most popular way to exchange and retrieve data, but since it is shared across a network, there is a danger of virus attacks and even a chance that personal information may be compromised. The constraints of healthcare requirements include data sharing, data access, data transmission, authenticity, and interoperability.

An EHR system (electronic health records) has replaced the conventional manual filing method, although it is expensive and labor-intensive. Following the introduction of EHR cloud-based systems to address the problems with EHR systems, however, these cloud-based systems still fell short in terms of encryption, data confidentiality, interoperability, and security standards. Every challenge the healthcare industry has, from interoperability to data security and transmission, might be addressed by blockchain technology.

**Figure 1:**
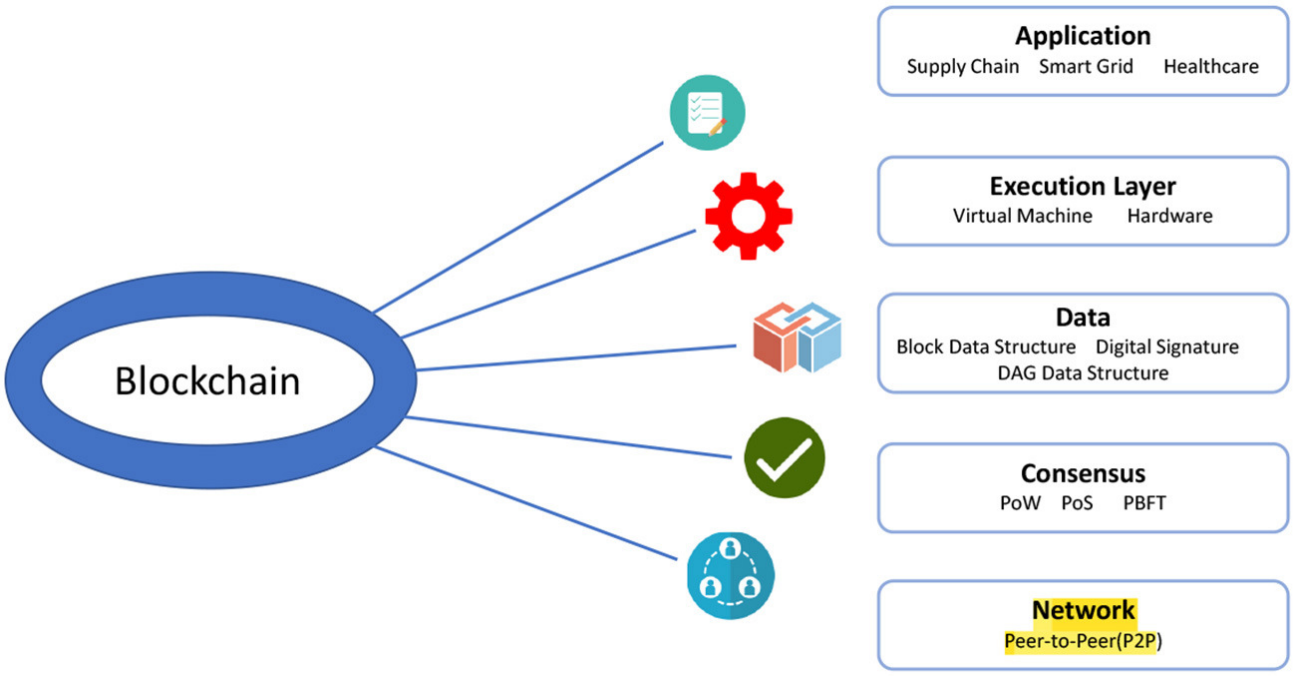
Architecture of blockchain.

Blockchain technology has several characteristics that include immutability, transparency, distributed ledgers, data security, authentication, and decentralization. This highlights why and how this technology is becoming increasingly popular across all industries, but especially in those like healthcare where issues with legitimacy, dependability, and security are especially problematic. The blockchain architecture is seen in Figure 1 and consists of three layers: an application layer, a transaction layer, and a network layer. Users will communicate with one another using blockchain applications in the application layer, and immutable transactions will be carried out in the transaction layer and on a global ledger. At the network layer, information exchange will take place on a P2P network.

## 2. Motivation

It is now well acknowledged and perhaps even evident that blockchain will most likely significantly disrupt the healthcare sector. Nevertheless, the authors tried to define the amount of relevance anticipated in as many ways as possible, to measure it, and to quickly characterize it in general terms.

**Figure 2:**
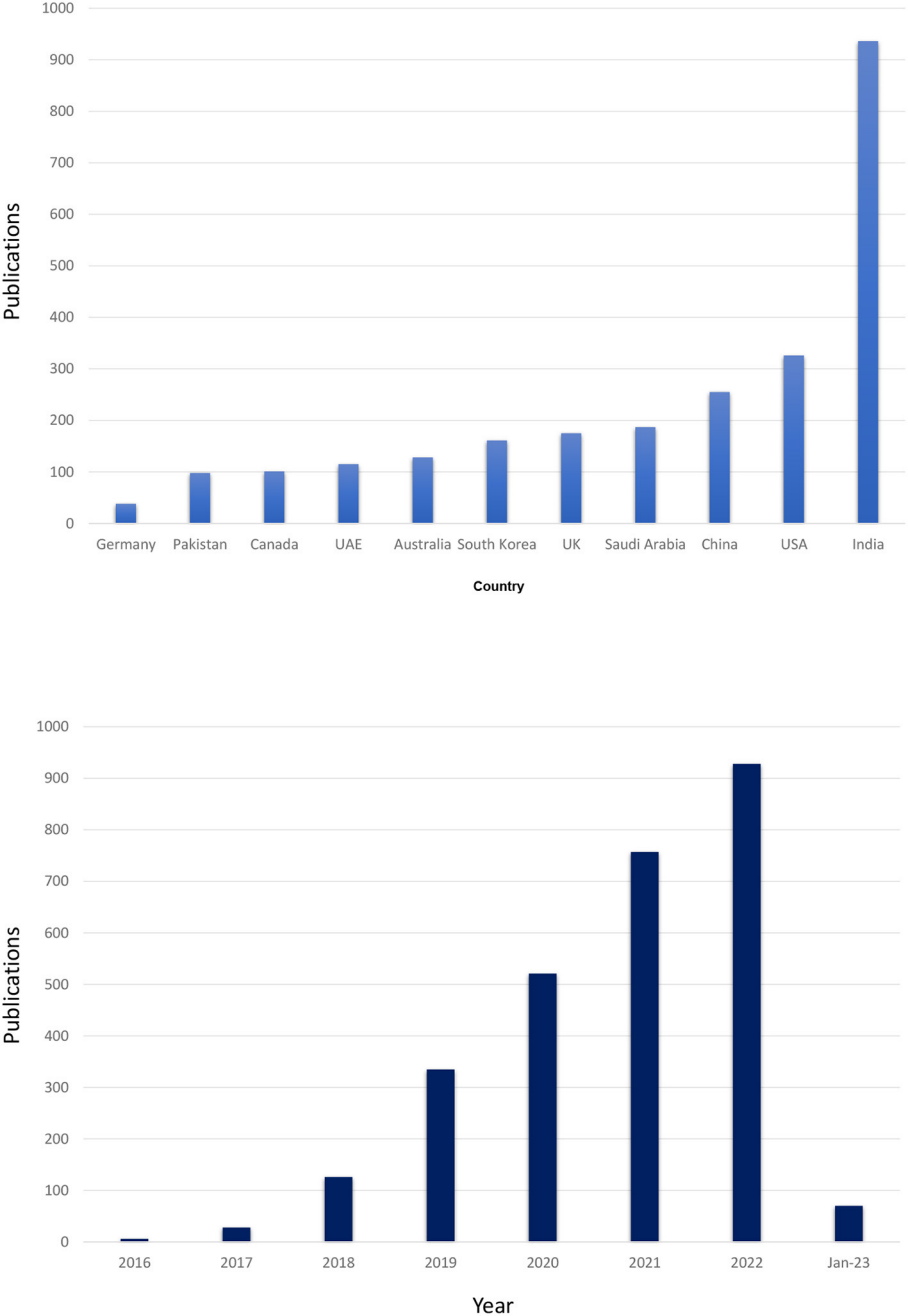
Publications per country over years.

**Figure 3:**
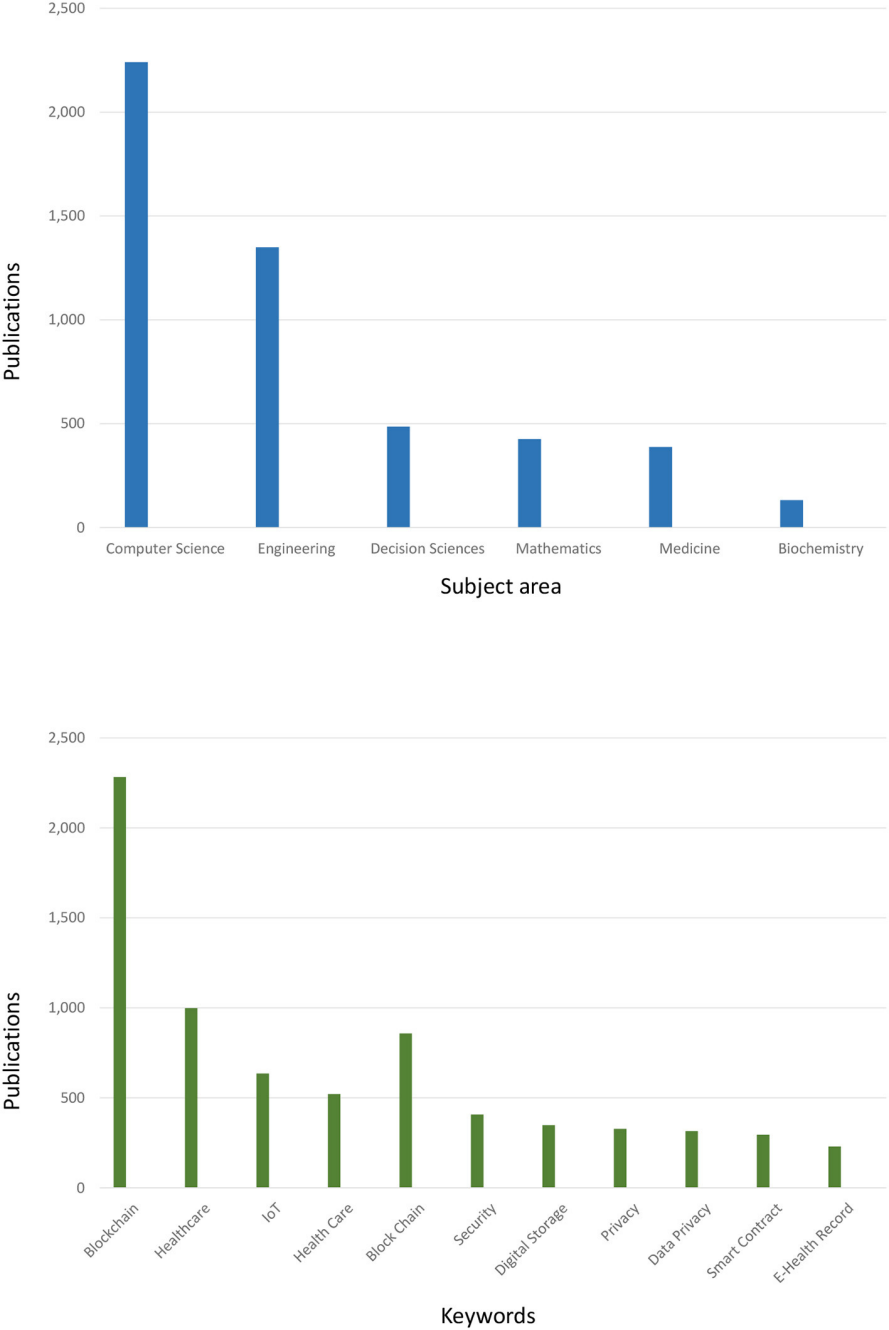
Publications in subject area and keywords.

In order to start with peer-reviewed papers, a search at Scopus was made in January 2022 for publications that had both “blockchain” and “healthcare” in their “Article” or “Abstract” or “Keyword” sections. This search returned a total of 1,776 documents (Figure 2). Figure 3 shows their distribution according to their country of origin, scientific field, keyword, and year of publication. The authors can clearly see the apparent quick growth in the number of relevant publications, as well as an intriguing keyword distribution where some keywords (like “security” and “privacy”) do not occur as frequently as expected. It is important to note that interest has extended globally, in addition to China and the USA. On January 2023, the authors ran the same query and received 2,828 documents in response. According to this, there are around three articles about blockchain technology in healthcare every day.

**Figure 4:**
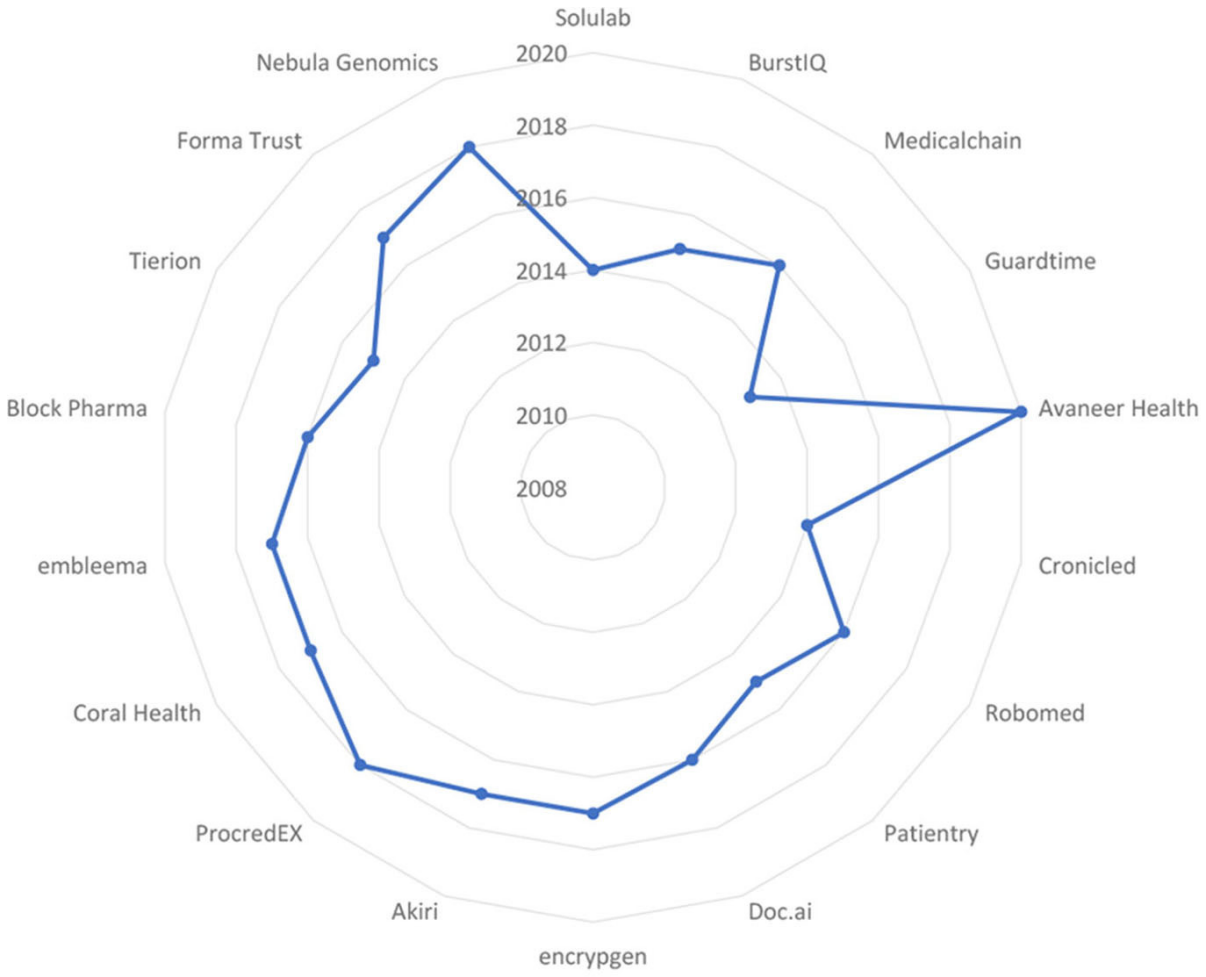
Time distribution of projects.

A similar search at Solulab turned up the 18 projects financed by independent companies under their Research and Innovation initiative. The acronyms for these R & D projects are provided below, followed by their titles, and they are ranked by Solulab according to how closely they adhere to the keywords. Presents a visual representation of the chronological distribution of these initiatives. These projects' time distribution is graphically shown in Figure 4.

BurstIQ: The organization uses blockchain to enhance the sharing and utilization of medical data.

SoluLab: Solulab uses blockchain to strengthen healthcare cybersecurity and to increase the sharing and use of medical data.

Medicalchain: The blockchain-based infrastructure used by Medicalchain keeps track of the source and safeguards patient identification.

Guardtime: Blockchain technology is used by Guardtime in cybersecurity applications, including healthcare.

Avaneer Health: Avaneer is a startup that seeks to use blockchain technology to improve healthcare efficiency. It is supported by Aetna, Anthem, and Cleveland Clinic. This is accomplished by utilizing a public ledger to provide improved claims processing, safe healthcare data transfers, and upgrade provider directories.

Chronicled: The usage of the Chronicled blockchain network ensures the secure delivery and thorough examination of drug supplies.

ProCredEx: ProCredEx has created a decentralized record system for healthcare credential information, ensuring that the data cannot be altered and remains permanently trackable. This enhances the effectiveness of intricate datasets, allowing for data customization to meet specific organizational requirements and the secure sharing of this data with authorized collaborators.

Robomed: Robomed captures patient data via blockchain and securely distributes it to the patient's healthcare professionals.

Patientory: The blockchain platform used by Patientory makes it possible to store and send sensitive medical data securely.

Doc.ai: The company uses artificial intelligence to decentralize medical data on the blockchain.

Encrypgen: It is now simpler to locate, share, save, and purchase genetic information because of the company's blockchain technology.

Coral Health: Coral Health uses blockchain to streamline administrative procedures, speed up the delivery of treatment, and enhance patient outcomes. The startup establishes faster connections between doctors, scientists, lab workers, and public health authorities by incorporating patients' records into DLT. To ensure that information and treatments are correct, Coral Health also uses smart contracts between patients and medical experts.

Embleema: Embleema is a tool for regulatory analytics and virtual experiments intended to accelerate the drug development process. Users are encouraged to provide their digital consent for the safe, unaltered acquisition of their medical data, which is subsequently recorded on the blockchain of Embleema and examined.

Blockpharma: Blockpharma provides a method to combat medication fraud and counterfeiting. Patients may find out whether they are taking fake medications using the company's app by scanning the supplier base and validating all points of shipping. Using a blockchain-based supply chain management system, Blockpharma claims it can screen out the 15% of medications that are fake worldwide.

Tierion: The blockchain of Tierion verifies all documentation, data, and pharmaceuticals to maintain a complete record of ownership. The company maintains evidence of ownership throughout a medical supply chain using timestamps and credentials.

FarmaTrust: The blockchain solutions from FarmaTrust may be used to manage prescriptions, validate the legitimacy of medical equipment, and protect patient data when they schedule vaccines and diagnostic tests. For example, the firm offers a service that prevents fraudulent pharmaceuticals from entering the supply chain and an app that allows customers to verify that their medications are authentic.

Nebula Genomics: Nebula Genomics is utilizing distributed ledger technology to eliminate extra fees and intermediaries in the genetic research industry. Annually, companies in the biotech and drug industries spend billions of dollars to get genetic data from other sources. While contributing to the development of a substantial genetic library, Nebula Genomics invites users to sell their encrypted genetic data securely.

## 3. Background and concepts

**Figure 5:**
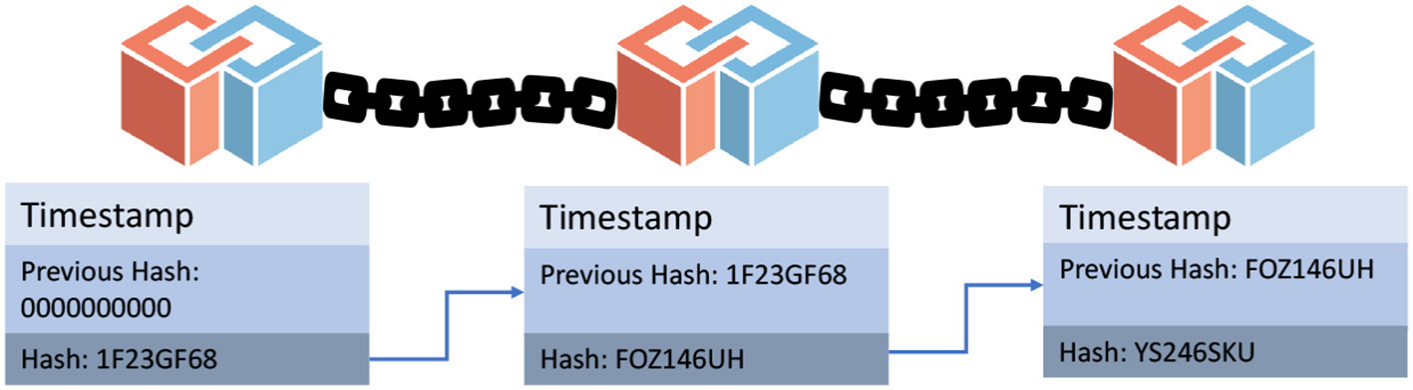
Representation of blocks in blockchain. Blockchain blocks include transaction details, including sender and recipient information, transaction size, and hash value. Blockchain is made up of a collection of interconnected blocks that are linked together using hash values.

In 2008 Satoshi Nakamoto implemented the cryptocurrency named Bitcoin based on the web white paper (1). This cryptocurrency works on open source technology and on the decentralized network in simple terms all nodes are connected mutually, and these nodes have the authority to leave and rejoin the network on demand and later receives the authentic record i.e., Proof of Work (PoW) referred to as the blockchain (1). To rejoin the network, they had to perform certain large computations to show evidence of their authentic members. PoW describes and gives proof of what happened when the particular nodes left the network. In cryptocurrency there may be a threat of a Sybil attack and this situation can be solved by claiming PoW from all nodes of the network which verifies transactions. The working of PoW can be understood by understanding the block of the bitcoin structure. The network consists of nodes that are nothing but participants and all of them have an identical ledger copy and these blocks of information are attached (2). These blocks consist of transaction data of both sender and receiver, the extent of the transaction, and hash value. Hash values are used to link the blocks, therefore Blockchain is a series of blocks tailored together as illustrated in Figure 5. It reflects, how blocks are linked together in blockchain. The order of blocks linked together will be determined by PoW consensus in Bitcoin. Bitcoins are chained using hashing. Changing the hash value will lead to the invalidation of a block. To validate, the block hash value needs to be recalculated. Bitcoin as being public blockchain technology is susceptible to security and privacy threats; this property is not acceptable for healthcare systems where data privacy is concerned. Bitcoin along with throughput is most desirable for building healthcare applications (3).

Blockchain is best characterized by a decentralized, immutable ledger that records data. It enables entities to communicate with one another without the requirement for a centralized, reliable middleman. The blockchain contains blocks of data, comprising sets of information that grow consistently. Once integrated into the blockchain, these blocks are linked to the preceding and subsequent blocks through cryptographic procedures. All parties can read, write, and modify these data records/blocks in the blockchain's original form. Decentralized transactions and data processing are made possible. These characteristics have made blockchain very popular for a variety of uses. Blockchain also supports smart contracts, fully independent contracts that may be executed without a central authority. As of right now, Ethereum is the blockchain that facilitates smart contracts.

### 3.1. What is blockchain?

#### 3.1.1. Key characteristics

Blockchain is decentralized, so nobody has full control over the data that is uploaded to it. Instead, a P2P network utilizing different consensus mechanisms approves the data that is uploaded to the blockchain. Persistence is another essential component of blockchain technology. Due to the distributed ledger's massive node storage, it is highly challenging to remove anything after it has been published onto the blockchain. Additionally, a lot of blockchains make use of the desirable potential anonymity (or pseudonymity) characteristic. By linking each block in a chain of blocks by containing the hash of the one before it, blockchains offer traceability and transparency. The Merkle tree-based organization of the blocks' transactions enables independent root-to-transaction verification (4, 5).

#### 3.1.2. Type of blockchains

Blockchains come in three basic forms: consortium, public, and private. They have a wide range of characteristics that influence who is able to read from, write to, and access data on the blockchain. All users have access to the data on a public chain, and anybody may contribute and make changes to the consensus and the core software. The two biggest cryptocurrencies, Bitcoin and Ethereum, which are categorized as public permissionless networks, are among the several cryptocurrencies that utilize the public blockchain. Because only a small number of carefully chosen groups of businesses have access to observe and take part in the consensus process, a consortium blockchain may be perceived as being somewhat centralized. A decentralized network that is frequently centralized makes up a private blockchain. A few nodes can connect to the network, and they are often under the supervision of one central authority (4, 6, 7). The definition and classification of the many blockchain types discussed here are still subject to debate. There is currently no widespread agreement on what defining characteristics and consensus procedures are necessary to refer to a piece of technology as “blockchain” (8). For the creation of decentralized applications, there are currently available blockchain frameworks and platforms (Dapps). The most well-known blockchain development platforms to date are Ethereum (decentralized platform) and Hyperledger (framework), both of which let programmers add new blockchain apps to current blockchains and construct new test nets using their protocols (9).

#### 3.1.3. Consensus algorithms

The procedure for approving data records on the decentralized ledger is a crucial component of blockchain technology. A distributed consensus method that checks the data entries accomplishes this. For this, a variety of consensus techniques have been put out and used; the three most popular ones are shown in Table 2 and are given below:

**Table 2:** Comparison of consensus mechanisms.

**Characteristics**	**PoV**	**PoS**	**PBFT**
Management of nodes	Accessible	Accessible	Permissioned
Usage of energy	High	Medium	Low
Tolerated an adversary power	< 25%	< 51%	33.3% faulty
Example	Bitcoin	Ethereum	Hyperledger

Proof of Work (PoW): PoW is the consensus system that is most closely connected to the blockchain because it is a part of Bitcoin. The process confirms the transaction and creates a new block for the blockchain. Miners compete in this procedure to finish the network transaction first. Competing is involved in mining. Miners receive incentives for successfully confirming a new block. This idea is supported by evidence of the significant electricity needed for Bitcoin mining, which is currently comparable to the requirements of a small nation (10).

Proof of Stake (PoS): With PoS, the node that will serve as an approving node is chosen based on its stake in the blockchain. When it comes to cryptocurrencies, a person's balance in a certain currency represents their investment. The “richest” node may, however, unjustly gain from this. Many hybrid PoS systems have been put out as a solution to this problem, where the approving node is selected using a mix of the stake and some randomization. The second-largest cryptocurrency, Ethereum, plans to transition to Proof of Stake from Proof of Work (4).

Practical Byzantine Fault Tolerance (PBFT): The underlying protocol of PBFT is a Byzantine agreement mechanism. This consensus procedure can't be used in a public blockchain since every node in PBFT must be known to the network, which places limitations on its use. Pre-prepared, prepared, and commit are the three separate stages of the PBFT consensus process. A node must get two-thirds of the votes from the other nodes in order to pass through the three phases. PBFT is now used by Hyperledger Fabric (11, 12).

#### 3.1.4. Smart contracts

Smart contracts are supported by blockchain infrastructures like Ethereum. These are executed automatically contracts with clauses that are written clearly into the source code. Smart contracts operate independently of any third parties or middlemen since they are automatically implemented based on these set clauses. A blockchain transaction may activate this smart contract feature, and the healthcare industry seems like an appealing use for it (7).

### 3.2. Significance of blockchain in healthcare industry

**Figure 6:**
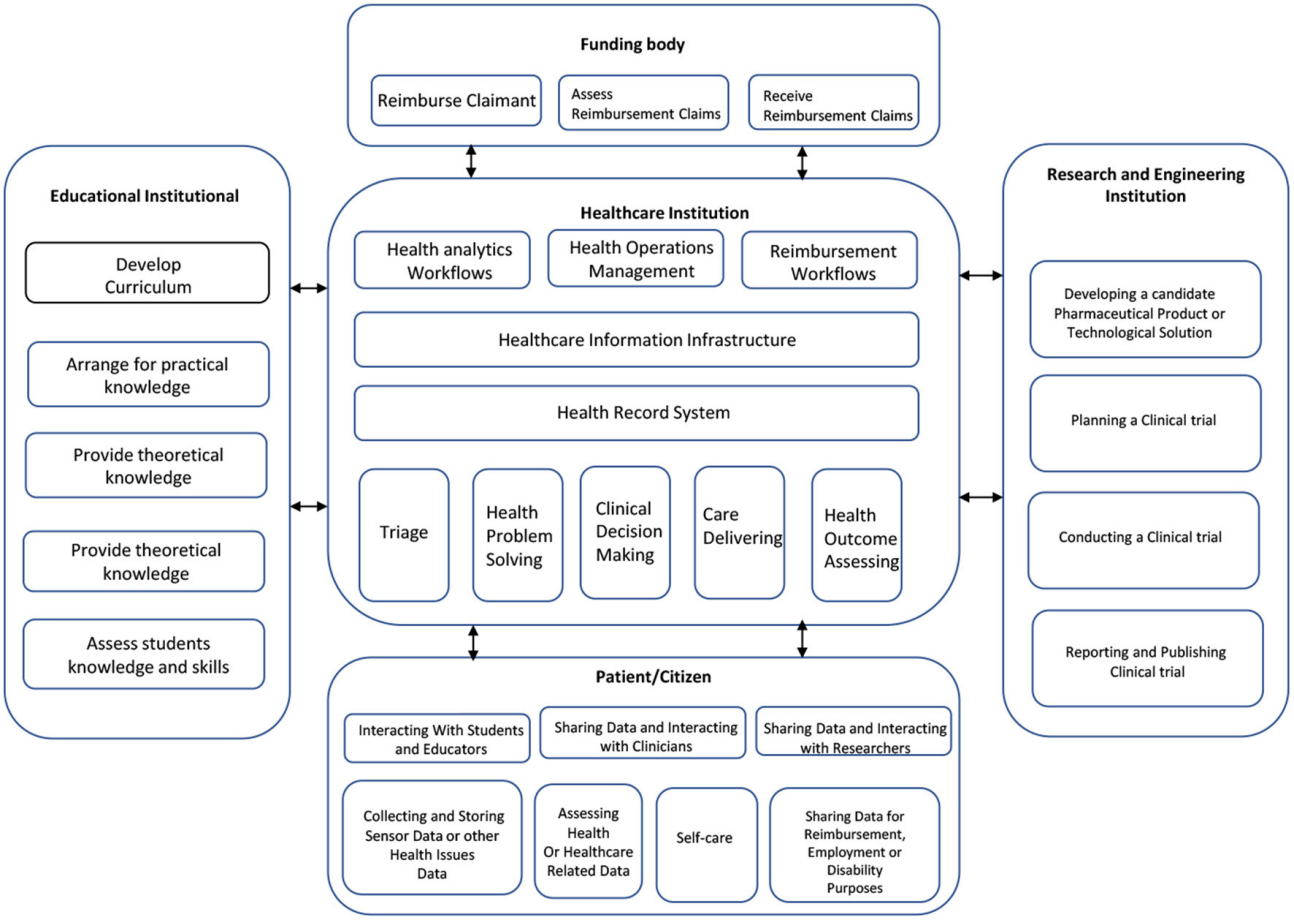
Map of healthcare industry.

Healthcare is a problem-driven, people- and data-intensive industry, and access to, updating, and trust in the information generated by its operations are essential for the sector's overall operations. According to a classification of healthcare operations into accident and emergency, health problem-solving, clinical decision-making, realization, and evaluation of knowledge-based care (Figure 6), it is essential to have a multidisciplinary team of healthcare professionals who treat patients with the best knowledge, technologies, and skills. To help students learn and develop their skill sets, the healthcare industry must work with educational institutions to give them access to patients and a training environment. In exchange, educational institutions give the industry skilled staff.

The healthcare sector faces a number of challenges, including data fragmentation, security and privacy issues, and interoperability problems caused by the usage of numerous standards in healthcare systems. By assuring secure and impenetrable data storage through a distributed ledger system, enabling secure data sharing to improve interoperability, and protecting patient privacy through data encryption, blockchain technology presents a potential solution. In the context of blockchain, various technologies have been implemented to address these issues, such as electronic health records (EHRs), health information exchanges (HIEs), and federated learning. While blockchain has great potential, it is important to understand that it is not a cure-all, and the healthcare sector is still exploring a variety of methods and technologies to meet its changing demands.

Health institutions must assist with access to experts, informants, test subjects, and samples when working with organizations and businesses for research and engineering purposes. Health institutions are required to help in the design, planning, execution, and analysis of the studies while taking part in prospective clinical trials. In exchange, the research and engineering organizations offer the healthcare industry the most recent information, practices, and technology. Therefore, the operations of health institutions are closely linked to those of organizations that educate health professionals and conduct biomedical research and engineering (Figure 6). The efficient sharing of patient-related information and evidence, along with reimbursement processes, necessitates the exchange of data between different institutions. It's crucial to safeguard the sensitive data patients entrust to healthcare organizations. Ensuring patients' privacy while sharing data with other entities in the healthcare network requires measures like access control, maintaining data origin, preserving data integrity, and enabling interoperability. The traditional way of implementing access control assumes a level of trust between data owners and the entities holding the data. These entities often manage access restrictions. To enhance the health of individuals and communities through collaborative data access, exchange, and utilization, a variety of information systems, devices, or applications need to seamlessly connect within and across organizational boundaries in a coordinated manner.

Data provenance refers to the origins and historical records of data sources. It can enhance transparency and reliability in electronic health records (EHRs) and foster consumer confidence in EHR software. According to Courtney and Ware, data integrity encompasses data quality and its expected standards. This means that meeting or surpassing these standards directly impacts data reliability. The demand for real-world data from businesses and research units is increasing within healthcare institutions. Simultaneously, the public's trust in healthcare organizations is waning due to instances of unlawful data sharing, widely publicized breaches, and private information theft. Another constraint is the existence of healthcare system mispractices that exploit the same trust level, involving issues like counterfeit medications, fraudulent personnel, and patients. Given this overall scenario, a reassessment and adoption of alternative strategies are imperative (13, 14).

## 4. Related works

In this study, the keywords such as “Blockchain in healthcare,” “healthcare record(s),” “healthcare system(s),” “healthcare and system and record(s),” “healthcare and blockchain,” and “healthcare and blockchain” were used to search the Scopus and Google Scholar databases for EHR-related literature. By limiting the inclusion of the research in these influential review publications, this manuscript provides a concise overview. In this study, the word “Healthcare” is used instead of “Healthcare,” as “Health care” refers to an industry or system that enables individuals to access the medical care they require (12, 15, 16).

People have expressed a desire to save their medical records as new technologies have emerged. For instance, research revealed that 87% of the health data that US citizens have gathered for themselves and their families are physical copies and that 42% of US citizens have done the same (17). EHR systems include several restrictions (like security) that make it challenging for users to exchange information. Rezaeibagha et al. (16) took into account the implications of security and privacy on EHR systems in their assessment of healthcare systems. They recognized a number of important aspects that affect information security and privacy, including encryption and scaling techniques, laws and regulations applicability, agreement and choice mechanisms, and integration and sharing of information. In the latest review to investigate the effectiveness of EHR systems, Afrizal et al. (18) explored the perspective of both individuals and an organization. Their research revealed organizational limitations, such as a lack of teamwork, inadequate executives, and a shortage of competent personnel. Aside from that, each person had their own limitations, such as a lack of computational resources and apprehension about novel technologies.

Modern technologies reduce the aforementioned restrictions. For instance, there are various ways to use blockchain to reduce hurdles in electronic health record systems (19). Blockchain is a distributed ledger technology that records network member transactions using immutable, reliable, and encrypted data (20). A system is referred to as a completely distributed system when no individual authority manages transaction operations that require the computing labor of several machines (21). UN's sustainable development goal may be greatly enhanced and achieved using blockchain, particularly in the healthcare sector (22). EHRs are one example of a public sector function that can be modernized with blockchain (23).

Zhang et al. (19) investigated the application of blockchain in healthcare systems using health scenarios that highlighted a patient-centric strategy in a framework for safe data sharing. They proposed adopting blockchain in seven different areas, including clinical documentation, patient-supervised cancer information, telemedicine treatment, patient verification, and health insurance disputes. In order to show how blockchain is related to patients' information-sharing behavior, authors focused on their health data. Even though Zhang et al. emphasized the benefits of utilizing it in health record administration, very few current works have offered a foundation for employing blockchain for patient health records. Homans et al. (24) built a blockchain-based management information system for electronic health records to solve security and privacy concerns. The ledger, database, committer, “orderer,” endorser, and client were suggested as the six components that make up the framework. Fan et al. did not concentrate on the concepts of privacy labor and digital money and left them for future investigations.

Griggs et al., Fan et al. (25), added to the work done by Fan et al., using Homans (24), by introducing a private blockchain to address privacy concerns in blockchain usage. Public and private blocks come in two varieties. A block is a complete record of every completed and pending transaction. A private blockchain can be a useful option in healthcare administration, according to Griggs et al., given the serious security issues surrounding personal information. Reduced opt-in rates might be the outcome of privacy problems in EHR systems.

The study proposes “PeNLP Parser,” a tool developed to extract and visualize exact geographic information about maternal, neonatal, and pediatric healthcare from unstructured data. The application extracts pertinent data and geolocations from the unstructured data using Natural Language Processing (NLP) techniques. By employing PeNLP Parser, healthcare providers and researchers can efficiently access and visualize essential geo-location data, enhancing their ability to make informed decisions and improve maternal and child healthcare services (26).

An integrated ontology is presented in the study by Patience et al. to aid in decision-making in the Maternal, Newborn, and Child Health (MNCH) sector. Context awareness is a feature of the ontology that enables it to take a variety of situational circumstances into account while offering decision help. By utilizing the integrated ontology, which can effectively analyze and understand data relevant to maternity, newborn, and child health to provide insightful analysis and suggestions for healthcare professionals and policymakers, the study seeks to improve decision-making processes in MNCH (27).

Sharma et al. (28) used the technique of the soft system to present qualitative evidence demonstrating that the usage of EHRs assisted with blockchain can increase patient engagement opt-in rates. They worked on the PHC strategy, which consists of a number of separate EHRs that are meant to be available to everyone in order to advance the healthcare system. They demonstrated how their suggested blockchain-based approach may boost patient and doctor trust in the sharing of medical records, while also enhancing the security and privacy of trustless PHC platforms.

The prospective impacts of blockchain on HIE were taken into account by Esmaeilzadeh et al. (29) their findings demonstrated that consumers are particularly planning complete blockchain-based privacy protection tools. Shahnaz et al. (30) provided a framework to reduce the scalability issue in the usage of blockchain in order to enable the adoption of blockchain in EHR. Blockchain-based healthcare systems have both beneficial and detrimental effects on patients and healthcare professionals, which presents new study opportunities (31).

The use of blockchain technology in the healthcare industry administration has lately been the subject of a number of studies, although its exact function in healthcare systems is yet unknown (32). To the best of the authors' knowledge, this is the only study to date that systematically examines the correlation between the intention of patients to share medical information and blockchain technology through mediating effects. The role of external incentives and security/privacy in the information system of a healthcare practitioner is also not well understood conceptually.

## 5. Applications of blockchain in healthcare

**Figure 7:**
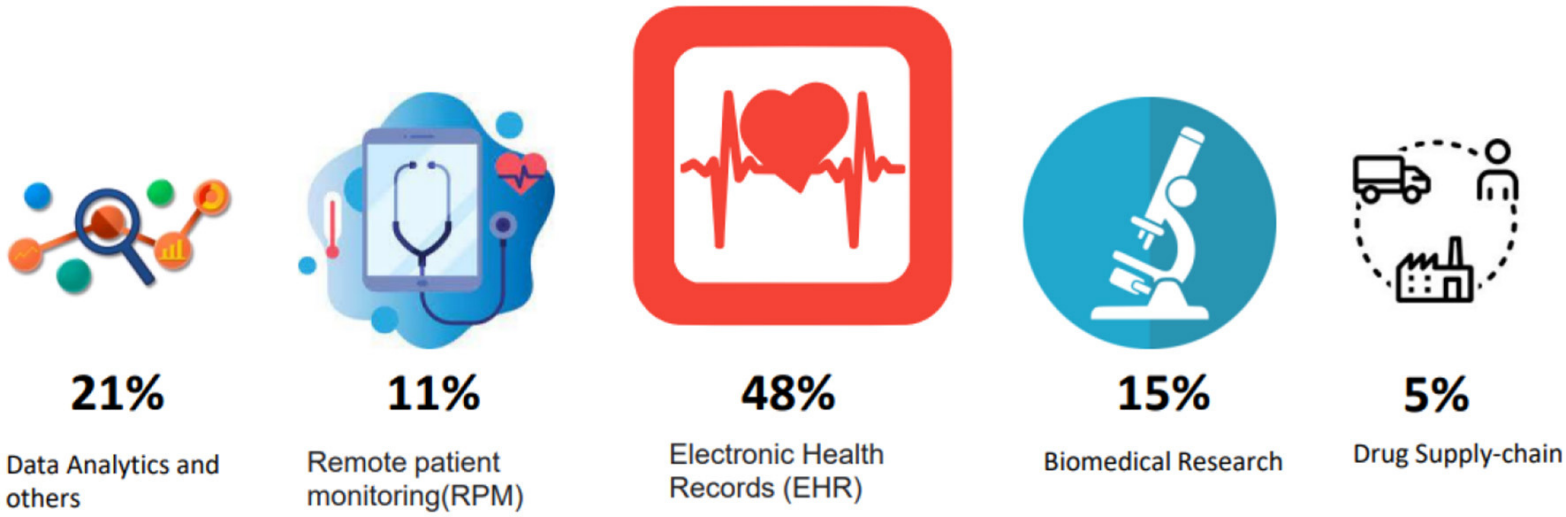
Emerging healthcare blockchain uses cases.

Blockchain technology possesses the potential to enhance the healthcare sector by prioritizing the patient within the system and enhancing the safeguarding, security, and seamless exchange of health information. In essence, the healthcare industry could undergo a substantial transformation through the widespread implementation of blockchain, resulting in comprehensive improvements in safety, security, and openness across all operations. Blockchain has the ability to improve things in this specific industry. It may perform a range of tasks, including controlling epidemics and safely encrypting patient data. Finally, by enabling secure data sharing between multiple healthcare systems with patient authorization, blockchain may enhance digital health (Figure 7).

### 5.1. Electronic health records

Blockchain technology could be employed to exchange and store patients' EHR. It may offer a supporting system for the exchange of health information that is safer, more transparent, and traceable. With the use of this technology, several data management systems that currently function in isolation might be linked to creating an EHR system that is both interconnected and functional. Therefore can patients and healthcare professionals easily access health information stored on the blockchain. It may be summed up in four easy steps:

1. The patient is examined by the doctor, who also registers the patient's report, lab findings, prescribed medications, and important comments in their current health information system. The patient's government-approved and accepted approved ID-related data fields are then transmitted to the blockchain using APIs. Here, a transaction is established.
2. Each transaction on the blockchain is verified and given a unique public key that would be stored on the blockchain.
3. Using the patient's decryption key, doctors and healthcare organizations may use APIs to build a query that retrieves the encrypted patient data.
4. Patients can give their doctor or the healthcare institution authorization to decrypt their data by giving them the private key, which serves as a password. The information is nonetheless encrypted for those without a secret key.

Asaph et al. introduced a decentralized system for managing medical records, aimed at handling electronic medical records (EMRs). In this system, MedRec provides patients with a comprehensive and reliable log of their medical history. This log is easily accessible and empowers patients with a better understanding of their medical past and any modifications to it, thus restoring their control over their medical information. The authors established a mechanism for patients to initiate sharing of their data across different medical entities using blockchain-based permission management.

MedRec's architecture enables specific permissions to be granted with a focus on maintaining confidentiality at a very detailed level. Additional constraints, such as setting time limits on viewing rights, can be placed within the various metadata segments that constitute a single medical record. These constraints can be independently communicated through smart contract provisions. By utilizing blockchain technology, the ledger ensures a transparent and traceable record of all medical interactions involving patients, doctors, and regulatory bodies (33).

### 5.2. Genomic data exchange platform

Platforms built on the blockchain aim to solve some of the biggest problems with governance, including the exchange of genetic data. The ultimate objective is to guarantee that organizations and people may exchange data with privacy-preserving algorithms that make it easier to adhere to moral and legal obligations. Even though most new platforms are still in their infancy, they might be regarded as advantageous since they provide fresh solutions to the governance issues associated with the sharing of genetic data. Notably, Blockchain represents more than a mere technological foundation; it introduces a novel approach to overseeing open networks that leverages the advantages of decentralized systems, market dynamics, and consumer genetics. As a result, the primary innovation in this context surpasses technological aspects, although it is facilitated by them. Networks built on blockchain hold the potential to amplify data volume while introducing fresh ownership models and fostering active user participation in data-sharing governance. Especially in the realm of blockchain-based solutions, there's the potential to automate data access control processes, thus enhancing transparency and the availability of genetic data. Similarly, the incorporation of smart contracts could significantly bolster the enforcement of access agreements. This effort is noteworthy as it instills confidence among researchers and data custodians that subsequent data uses will adhere to the specified terms and conditions (Table 3).

**Table 3:** Applications/delivery functions of blockchain in healthcare.

**S.no**	**Application**	**References**
1	Access control	(34–37)
		(38–41)
		(42–46)
		(25, 33, 47–49)
		(50–53)
		(54–59)
		(45, 60–64)
2	Privacy	(48, 65–74)
3	Distributed computing	(75–77)
	HIV	(78)
	LMIC	(79)
	Cancer	(73, 80)
	Diabetes	(81)
	Insurance	(82)
	Dermatology	(83)
	Clinical trial	(46, 77, 84, 85)
	Supply chain	(86–88)
	Genetics data	(89)
	Communication	(51)
	Medical imaging	(78, 90)
	Software design	(81, 91)
	Pharmaceuticals	(92)
	Health education	(47)
	Blood management	(93)
	Radiology oncology	(91, 94, 95)
	Patient-centered care	(85, 96–98)
	National Health system	(62)
4	Service delivery	
	Dyslexia	(99)
	Remote care	(100)
	Dental care	(99)
	DNA compression	(101, 102)
	Hemoglobin test (HbAlc)	(103)
	Arrhythmia classification	(68)

Furthermore, effective implementation of the Blockchain-driven solution has the potential to reshape cultural norms around data sharing. This could result in a shift away from the dominance of public and commercial genetic test providers in controlling the dissemination of genetic information. This shift would empower patients and individuals to play a more influential role in the data-sharing landscape. Blockchain technology holds the capacity to establish novel shared resources that bridge the gap between market-driven dynamics and public resources. To initiate this transformation, substantial efforts in education, incentive design, ownership structure, and collaborative governance may be required. The aim of blockchain-based platforms is to empower patients and citizens to have agency over their data and participate in data sharing. Nonetheless, it's important to note that legal frameworks are still essential for the success of Blockchain-based solutions.

There are crucial scenarios where self-regulation might fall short, such as when assigning value to specific genetic datasets and determining ownership rights. To ensure that regulations align with the best interests of scientific advancement, society, and innovation, a thorough evaluation of the broader impact of such regulations within the realm of biomedical research is of utmost importance (104).

### 5.3. Medical imaging

Many scientists have been working on creating a feasible method for storing and distributing medical images in the realm of healthcare in recent years. The use of centralized cloud-based data centers in current practices raises privacy problems when exchanging information across a network, increases maintenance expenses, and necessitates vast storage capacity. The chain of transactions on the blockchain simply contains a list of the key owners who are authorized to view each research; no medical pictures are kept there. The image recipient must issue a signed request to the URL endpoint of the imaging source that generated the research before the actual image transfer can take place. Any person or organization that the owner (patient) who has granted permission to obtain this specific imaging study may be considered the requesting entity. The authors made use of the already existing work by the Integrating the Healthcare Enterprise (IHE) effort, which has established the ITI-43 transaction as a standard form for document retrieval across domains. The image source certifies the validity of the signature, confirms that the repository Unique ID specifies its own public key, confirms that the hashed UID matches to a study it previously released for the patient, and confirms—via the blockchain—that the patient has authorized the requester access to these images. If every requirement is met, the source sends back an ITI-43 response that includes the imaging study. To avoid eavesdropping, the request and the response are both sent via a secure channel at the transport layer (62, 105).

### 5.4. Pharmaceutical and drug discovery

Pharmaceutical research and development encompass a comprehensive journey, spanning multiple years dedicated to drug discovery, drug development, and regulatory approval within the pharmaceutical supply chain. However, drug counterfeiting occurs when drug producers and regulatory agencies conceal, lack control over, or use obsolete information on the supply of pharmaceuticals. This information results in the production, marketing, and use of fake pharmaceuticals. In situations like these, when data security and privacy protection are top priorities, blockchain is the most appropriate technology. It demonstrates the reliability of medical treatment for the people and the safety of pharmaceuticals sold on the market by utilizing current, genuine digital technologies. When considering the potential uses of blockchain technology in the sector, the pharmaceutical supply chain offers a convincing example: Pharmaceutical drugs are created and produced in specialized facilities before being routinely distributed to wholesalers and eventually patients. A possible way to improve medication research and secure the dependability of the pharmaceutical supply chain is through the incorporation of blockchain technology. The whole drug development process is facilitated and managed by this technology thanks to features like distributed ledgers, smart contracts, asset transfers, and proof of work (Table 4).

**Table 4:** Applications of blockchain in healthcare and related technology used.

**S.no**	**References**	**Application**	**Technology**
1	Zhang and Lin (34), Thwin and Vasupongayya (106), Yue et al. (107)	Personal Health Information (PHI)	Proxy re-encryption technique, private blockchain, and consortium blockchain
2	Patel (62), Roehrs et al. (98)	Personal Health Records (PHR)	Distributed P2P network system
3	Xia et al. (96), Badr et al. (38), Alexaki et al. (42),		
	Tian et al. (63), Pham et al. (102), Rouhani et al. (108)	Parallel Healthcare system (PHSs)	Hyperledger framework, Ethereum,
	Li et al. (109), Theodouli et al. (110)		Cryptographic functions, and smart contracts
4	Al Omar et al. (111)	Healthcare System (e-health)	Body sensor network
5	Zhao et al. (112)	Remote Patient Monitoring (RPM)	Loosely coupled Blockchain, off-chain storage, and on-chain verification.
6	Jiang et al. (88)	Remote Healthcare system (RHS)	MAM module of the IOTA protocol
7	Alamoodi et al. (113)		BSN, Sensor Data Provider, permissioned,
	Brogan et al. (114)	Remote Patient Monitoring (RPM) in IoT	consortium-managed blockchain, Private key,
	Uddin et al. (37)		public key, and Healthcare Provider Interface
8	Griggs et al. (64), Dwivedi et al. (115)	EHR in a cloud environment	Constrained Goal Model (CGM)
9	Wehbe et al. (116)	Medical insurance storage	Blockchain technology
10	Wang and Song (36)	Multi-site Clinical Trials	Artificial system modeling
11	Zhou et al. (85)	Telecare Medical Information System	Hyperledger Fabric
12	Wang et al. (84)	Healthcare Information Exchange	Blockchain and cloud
13	Choudhury et al. (117)	Medical Data Sharing in Cloud Environment	Blockchain

Despite its potential benefits, the influx of counterfeit and substandard pharmaceuticals into the legitimate supply chain poses a significant threat to public health. However, blockchain technology holds the promise of mitigating these risks and improving current systems. As the acceptance of blockchain technology becomes widespread, its capacity to revolutionize intercompany interactions is evident. While industries are only beginning to grasp its potential implications, it's essential to recognize that its applications span beyond specific sectors.

The full extent of the impact this transformative technology will have on the global landscape will become apparent over the years. As blockchain technology gains traction, its potential to reshape business interactions becomes increasingly apparent.

### 5.5. Remote patient monitoring in IoT

The utilization of the Internet of Things (IoT) and Blockchain advancements has found extensive application in various domains, such as remote patient monitoring (RPM). There has been swift development in crafting wearable medical devices within the IoT framework, equipped with diverse functionalities that facilitate the collection and analysis of live sensory data from patients. Data from IoT devices is gathered, analyzed, and stored centrally. However, there may be a number of drawbacks to this centralization, such as single-point failure, data manipulation, privacy concerns, etc. Blockchain's decentralized design can be used to solve these issues. Consequently, using IoT and blockchain to create a smart RPM system is a viable option. RPM is effective for treating a wide range of medical diseases, including diabetes, pediatrics, prenatal care, hypertension, and post-operative treatment. Patients can monitor their health at home with medical tools including blood pressure cuffs for hypertension, pulse oximeters for blood oxygen monitoring, glucometers for blood sugar levels, ECG machines for heart patients, and activity trackers. When one of these devices is attached to a patient, the health readings are automatically taken. The readings are then sent to the healthcare professionals who may monitor for health trends, and changes in conditions, and even be alerted when a patient's condition is likely to deteriorate using danger warnings.

### 5.6. Parallel Healthcare systems

The authors provided a paradigm for ACP-based Parallel Healthcare systems (PHSs) to improve the precision of diagnosis and efficacy of treatment. PHS uses computerized testing to analyze and evaluate a variety of medical prescriptions, simulating real-world healthcare systems to represent and reflect patient conditions, infections, and prescriptions. Healthcare operations for both humans and machines use real-time advancement as well as data-driven decision support in system arrangement. Additionally, they combined the recently developed blockchain-based PHS, which utilizes a consortium blockchain to interconnect patients, hospitals, healthcare organizations, and societies for critical health data interchange, medical record review, and accessibility to treatment (84).

### 5.7. Medical body sensor network

In addition to concurrent wireless technologies like wireless personal area networks, WBANs, and WPANs offer a lot of promise in healthcare monitoring systems to assess particular vital data and also to give location-based data (WPANs). A high incidence of both diagnostic and therapeutic studies is being driven by the expanding selection of wearable and subcutaneous medical equipment and their incorporation with wireless sensors. The development of Wireless Body Area Networks has been facilitated by the growing use of wireless networks and the ongoing shrinking of electrical invasive/non-invasive devices (WBANs). A WBAN allows for continuous patient health monitoring without interfering with the patient's regular daily activities. Numerous technologies have demonstrated their effectiveness in enabling WBANs applications by meeting their unique quality of service (QoS) needs, such as remote monitoring, biofeedback, and assisted living. It might be difficult to choose the best technology for a medical application because there are so many technologies that are now accessible.

### 5.8. Personal Health Records

A Personal Health Records (PHR) is a medical file where a patient manages their own health records as well as other information related to their healthcare. It is an electronic tool that enables people to manage their healthcare information securely. A patient may maintain and share their health information via an electronic PHR, which is secure software. Information entered by the patient from other sources, such as pharmacies and healthcare providers, may also be included. The medical professionals who assisted in the treatment processes might be held accountable for the patient's disease. PHRs can be stored electronically or using a computer program. The sort of information that each individual may access can be managed by users using PHR.

PHR aims to provide an accurate, online-accessible summary of a patient's medical history. Lab reports, patient-reported outcomes, and other information could be included in the PHR. The phrase first appeared in usage between 1956 and 1978. It was first used in paper-based and digital systems.

People think that PHR and EHR are similar. However, this is not true. Doctors maintain an electronic health record (EHR). Hospitals, pharmacies, and doctors' offices can all create personal health records. Its main goal is to give patients the tools they need to manage their information. PHR calls for all the tools necessary for you to manage your health with the guidance of your doctors. It includes information such as doctor's names, medicine allergies, family history, sickness dates, and extra dosages.

### 5.9. Drug supply chain

At the moment, counterfeit drugs are pharmacology's primary problem. Health Research Funding estimates that 10%–30% of medicines in underdeveloped nations are bogus. The effects that counterfeit pharmaceuticals cause are not just different from those of traditional drugs; they also have distinct consequences on human health. According to the World Health Organization, around 30% of the medications marketed in Africa, Asia, and Latin America are unfortunately fake. In underdeveloped nations, where one in every ten medicines could be fake or don't follow drug standards, this issue is regrettably becoming worse. Monitoring drug safety has become more difficult as a result of the rise of online pharmacies. These medications run via a more convoluted, dispersed supply chain, making it more challenging to detect fakes and providing possibilities for fake pharmaceuticals to penetrate the real supply chain. Concern over the security of the drug supply chain has increased within the public health community, a process that affects everyone. Public health, a process that involves everyone, has grown more concerned about the safety of the drug supply chain. Transparency might make medication supply chain surveillance and inspection considerably more effective and accessible. Laws and regulations pertaining to blockchain are currently being developed because the technology is in its genesis stage. Even blockchain technology itself is evolving (e.g., “smart contracts,” “Blockchain 2.0”), therefore more regulatory effect analyses and system simulation stress testing will be required in the future, along with engagement with key stakeholders, to undertake the cost-benefit analysis.

### 5.10. Fraud detection

The healthcare industry and public entities are very concerned about medical insurance fraud. The cost of healthcare fraud was reported as a loss to health insurance companies in the United States on an annual basis in the tens of billions. The patient's health is in danger from some types of fraud. This happens because the mechanism used to manually process medical insurance claims frequently fails to consider some stakeholders' consent throughout the assertion validation procedure. Blockchain is a peer-to-peer decentralized technology that can enable the secure, open, and unchangeable validation of medical claims.

Blockchain will be applied to Electronic Health Information (EHR) to address issues of security and privacy; by integrating blockchain with EHR, patients can effectively manage and save their records. Dissemination of patient data will be handled confidentially by blockchain using the blockchain medical records can be securely audited. With the blockchain, clinical data sharing will be securely and effectively managed with the help of blockchain. Blockchain enables IoT to provide a range of services, including Remote Patient Monitoring. Blockchain-based Remote Patient Monitoring will be maintained confidently. Health insurance companies are adapting blockchains to monitor false insurance claims made by patients. Various applications of blockchain and related technologies are listed in Table 2. Applications and delivery functions are listed in Table 5. Blockchain will be used in the pharmaceutical industry to address challenges like:

- Clinical data sharing.
- Supply chain of drugs.
- To manage clinical trials.
- Prescription management etc.

**Table 5:** Blockchain frameworks in the healthcare domain.

**Feature**	**Bitcoin**	**Ethereum**	**Hyperledger**
Security	Public	Private	Private
Speed (transactions/s)	7	15	3,000
Scalability	Low	Low	High
Cost of transaction	Low	Low	Moderate
Need of cryptocurrency	Yes	yes	no

## 6. Use cases of blockchain-assisted decentralized applications

Blockchain technology is still in its adolescence stages, and even when prototype apps are created, they may serve just experimental or least functional objectives. However, some publications give implementation information for programs that have been created for specific use cases. Examples of such apps for the EMR use cases are, Hyperledger Fabric-based Healthchain (118), Acile (119), and MedRec (33), both of which were created on the Ethereum platform and Other instances include MeDShare (96), BlockHIE (88), FHIRChain (81), and MedBlock (25).

The administration of the pharmaceutical and drug supply chain, biomedical education and research, handling health insurance claims, and remote patient monitoring are examples of other blockchain use cases that have been discussed. However, there are also potential blockchain use cases that are still conceptual and do not yet have prototypes, such as the usage of blockchain in legal medicine (120).

## 7. Constraints of blockchain-assisted decentralized apps

The development of blockchain-assisted applications has been slowed by a variety of issues, including interoperability, security and privacy, scalability, speed, and patient involvement. Due to the lack of an open standard, it may be challenging for applications made by different manufacturers or on technology to interact with one another. This creates an interoperability dilemma. Consider taking a look at the other RPM apps: one was programmed on the Ethereum network, and the other was on the Permissioned Blockchain platform. Information exchange between these two systems would be difficult because, despite the encryption mechanisms used, it may still be possible to determine a patient's identity on a public blockchain by correlating enough data that are pertinent to that patient, blockchain-assisted healthcare apps have received criticism for their lack of security and privacy. Additionally, there is a chance that security flaws brought on by hostile deliberate attacks launched against the healthcare blockchain by criminal groups or even governmental entities might compromise patients' privacy. Various cryptocurrencies' blockchain networks have allegedly been the victim of many hacks. The private keys utilized by the blockchain for the cryptographed data are also prone to hacking, which might give unapproved individuals permission to the stored medical data. How well the immutability aspect of blockchain will function with the “right to be forgotten” clause of the EU GDPR, which specifies that users have the right to request the complete deletion of their personal data, raises further doubts. When a patient's clinical history is wiped away, it may be problematic since data once synced on the blockchain may not be altered or changed because of its immutability. Blockchain-based healthcare systems have major scalability challenges, especially in light of the amount of data involved. It is not advisable to put a vast quantity of biological data on the blockchain since doing so will always result in a severe performance hit. There is also the question of speed, since processing with blockchain may result in significant delays. For instance, the current validation technique used by the Ethereum blockchain platform involves participation from each network node. This results in a sizable processing lag, particularly when the input file is substantial. The management of medical records on the blockchain presents another challenge, particularly the inclusion of patients. It's likely that patients won't be able to or desire to get engaged in the processing of their medical data, primarily individuals of younger or elder age.

## 8. Addressing challenges

There are several constraints and limitations over how blockchain can be implemented in health IT systems, and many methods are being put up to get around these restrictions. For example, it is advised that encrypted health data be stored “off-chain,” with just a limited amount of knowledge about the medical record and how to access it, in contemplation of the scalability issue. Thus, the “right to be forgotten” concern raised by the GDPR is similarly addressed. Although the connection to the medical data stored upon that distributed ledger cannot be unpublished, the particular medical data retained off-chain may be removed permanently. This countermeasure has a number of disadvantages, along with a gradual decrease in the built-in redundancy of the blockchain, which increases data availability. To better safeguard the data and maintain patient privacy, healthcare apps use permissioned blockchains, such as the consortium blockchain, instead of the permissionless, public blockchain. Additionally, by utilizing a strong software development strategy and all existing security safeguards during code development, many security vulnerabilities may be addressed. There are procedures in place on blockchains with permits for the healthcare sector that enable the rectification of transactions that go completely bonkers.

## 9. Future research

The application of distributed ledger technologies like blockchain in the healthcare industry is still in its premature stage, thus researchers must develop more proofs-of-concept and prototypes. This will aid scientists in their efforts to comprehend and advance technology as it pertains to healthcare systems. It is necessary to construct and test a number of the suggested principles, concepts, methods, and architectural designs in order to examine their merits and drawbacks. To check interoperability across diverse blockchain applications, open standards are necessary. Currently, the focus is on evaluating the capabilities of blockchain prototypes to demonstrate principles. Prior to blockchain becoming fully operational in healthcare systems, open protocols for compatibility must be developed. Researchers must immediately begin researching the issues with interoperability and standardization practices. Currently, there is a standards body (ISO/TC 307) where researchers may propose their concepts.

## 10. Conclusion

Blockchain technology has evolved since it was first used in Bitcoin to become a general-purpose technology with uses in many other sectors, including healthcare. The authors conducted a systematic review, employing the systematic mapping study methodology, to create a comprehensive overview of relevant research. This was done to gain insights into the current status of blockchain technology utilization in the healthcare sector. The study aimed to achieve several specific objectives: identification of healthcare applications utilizing blockchain technology, examination of exemplary apps developed for these applications, exploration of challenges and limitations linked to blockchain-based healthcare apps, analysis of the methodologies employed in creating such apps, and identification of potential avenues for future research. Through a meticulous search and selection process, the team identified 136 papers. These papers were subsequently scrutinized by the authors to address the research inquiries at hand.

The study we conducted revealed various healthcare applications for blockchain technology, including governing electronic medical records, overseeing the pharmaceutical and drug supply chain, advancing biotech research and education, enabling remote patient monitoring, and facilitating healthcare information analytics. To achieve these goals, different blockchain development approaches such as permissioned blockchains, off-chain storage, and smart contracts have been employed.

However, further investigation is necessary to refine, evaluate, and fully grasp the potential of blockchain technology in the healthcare sector. There is also a need for additional research to support ongoing endeavors aimed at resolving challenges related to scalability, latency, interoperability, confidentiality, and security in the implementation of blockchain-based healthcare solutions.

## Data availability statement

The original contributions presented in the study are included in the article/supplementary material, further inquiries can be directed to the corresponding author.

## Author contributions

SS and YS focused on Blockchain and its practical applications. YH, MJ, and OV dedicated their efforts to the effective implementation of blockchain in the healthcare sector. All authors have reviewed and approved the final version of the manuscript for publication.
